# In Vitro Assessment of Anticoccidials: Methods and Molecules

**DOI:** 10.3390/ani11071962

**Published:** 2021-06-30

**Authors:** Martina Felici, Benedetta Tugnoli, Andrea Piva, Ester Grilli

**Affiliations:** 1 Dipartimento di Scienze Mediche Veterinarie (DIMEVET), Università di Bologna, Via Tolara di Sopra 50, 40064 Ozzano dell’Emilia, Italy; martina.felici3@unibo.it (M.F.); andrea.piva@unibo.it (A.P.); 2 Vetagro S.p.A., Via Porro 2, 42124 Reggio Emilia, Italy; benedetta.tugnoli@vetagro.com; 3 Vetagro, Inc., 116 W. Jackson Blvd., Suite #320, Chicago, IL 60604, USA

**Keywords:** *Eimeria*, poultry, in vitro, cell culture, botanicals, essential oils, invasion assay

## Abstract

**Simple Summary:**

Coccidiosis is a major problem in poultry production, leading to significant economic losses. Due to the outbreak of resistance to the available treatments, research is focusing on finding new molecules that work against the pathogen. Botanical compounds represent promising alternatives, but reliable in vitro tests are needed for their screening and to understand their mechanism of action. Research in vitro involves studies on the environmental phase of the parasite and studies on the endogenous development, which occurs inside the host cells and that requires cell cultures or in ovo models to be studied. This review aims to summarize the protocols that have been successfully applied so far, as well as to suggest potential cues to improve research on this field. Moreover, as the surge of botanicals as anticoccidial molecules is on the rise, the intent is to provide an overview of the methods to assess their effectiveness in vitro in comparison with conventional drugs.

**Abstract:**

Avian coccidiosis is a disease causing considerable economic losses in the poultry industry. It is caused by *Eimeria* spp., protozoan parasites characterized by an exogenous–endogenous lifecycle. In vitro research on these pathogens is very complicated and lacks standardization. This review provides a description of the main in vitro protocols so far assessed focusing on the exogenous phase, with oocyst viability and sporulation assays, and on the endogenous phase, with invasion and developmental assays in cell cultures and in ovo. An overview of these in vitro applications to screen both old and new remedies and to understand the relative mode of action is also discussed.

## 1. Introduction

Coccidiosis is one of the main parasitic diseases in poultry production and it leads to significant economic losses, estimated to be up to USD 13 billion per year [1]. *Eimeria* spp., etiological agent of this illness, belong to the genus *Eimeria,* family Eimeriidae and phylum Apicomplexa. They are microscopic, spore-forming protozoan parasites that replicate inside the host enterocytes, causing severe diarrhea, weight loss, and, in some cases, sudden death. *Eimeria* infection can also facilitate other opportunistic pathogens, aggravating the clinical status of the animal. Field cases are usually characterized by various species at the same time, and the seven species mainly involved in the disease are *Eimeria acervulina, Eimeria brunetti, Eimeria maxima, Eimeria mitis, Eimeria necatrix, Eimeria praecox* and *Eimeria tenella;* they differ from each other in terms of dimension, severity, and site of the lesions [2,3]. *Eimeria mivati* and *Eimeria hagani* have been previously described, but they are considered nomina dubia by the majority of coccidiologists [4].

A good prevention and treatment of the disease is necessary, and it involves anticoccidial drugs such as ionophores and synthetic compounds and vaccines.

Ionophores and some synthetic anticoccidials are licensed in the USA as antibiotics and in the EU as zootechnical feed additives under regulation 1831/2003/EC for poultry species, and their use does not require a veterinary prescription [5]. However, the development of coccidial and bacterial resistance due to their extensive use, feed cross-contamination, and poorly elucidated interactions with other drugs has raised concerns about these treatments [6].

However, the need to limit the outspread of coccidiosis forces the industry to rely on these compounds, as, by now, they are the only available remedy. Rotation programs are often adopted to reduce the spread of resistance in intensive broiler farms, but drugs with novel modes of action will be necessary in the future [5]. Research on alternative solutions has recently focused on botanical compounds and natural remedies, as they interfere with various stages of the parasite development [7].

Botanicals and nature-identical compounds (NICs) are substances deriving from a part of a plant, from its essential oils (EOs), or from chemically synthetized plant molecules, and they have a well-known antimicrobial and immunomodulatory action [8]. New compounds are often tested in in vivo trials: those procedures are quite expensive, time-consuming, and require the sacrifice of many animals, so in vitro screening methods for the identification of anticoccidial agents would be advantageous [9,10]. Moreover, ethical concern for animal welfare is pushing researchers to adopt measures to replace, where possible, or reduce the use of animals for scientific purposes, in full agreement with the “3Rs guidelines” [11]. In vivo studies can only provide an overview of the general benefits of a treatment against coccidiosis, but the key processes that make it effective should be studied in vitro, too. In vitro research allows a detailed step-by-step analysis of the mode of action of a treatment, which is essential for pathogens like coccidia, characterized by a multiphasic life cycle, in order to understand the actual target of the molecule [12]. This review aims to summarize the protocols that have been successfully applied so far and the achieved results, and to suggest potential cues to improve research on this field.

## 2. *Eimeria* Life Cycle, Mechanism of Invasion, and Tools for In Vitro Research

**Figure 1:**
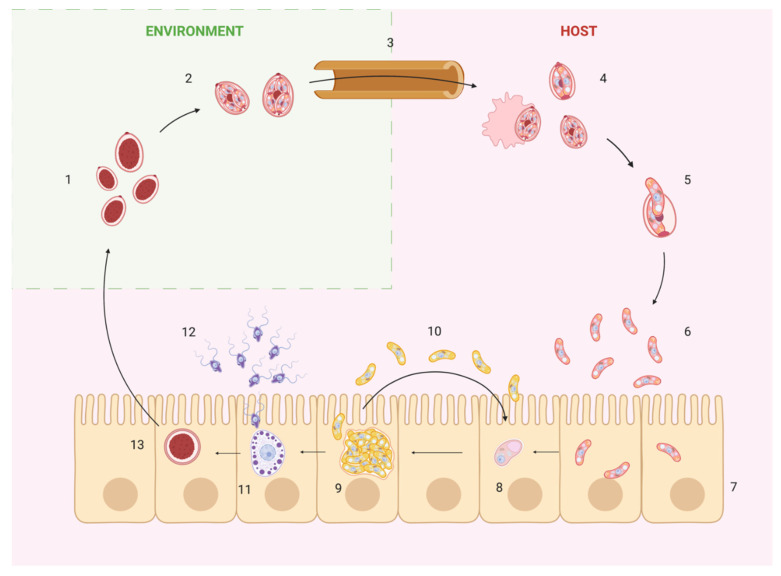
Schematic representation of coccidian life cycle. Numbers indicate the developmental stages of *Eimeria* spp. and the host structures involved: (1) unsporulated oocysts; (2) sporulated oocysts; (3) gastrointestinal tract; (4) mechanical rupture of oocysts and sporocyst release; (5) excystation of sporozoites; (6) released sporozoites; (7) enterocytes; (8) trophozoite; (9) mature schizont; (10) merozoites and re-infection of enterocytes; (11) macrogamete; (12) microgamete; (13) zygote.

The life cycle of *Eimeria* species alternates between an exogenous phase, which occurs in the environment, and an endogenous one, which occurs inside the gastrointestinal tract of the host (Figure 1).

Unsporulated oocysts are shed into the environment through feces; they contain a single undifferentiated cytoplasmatic mass surrounded by a thick protective wall [13]. At appropriate conditions of temperature (24–28 °C), oxygen supply, and humidity (40–80%), sporulation occurs by meiotic division [2]: during this process the cytoplasm divides in four sub-masses called sporocysts, which develop another protective wall. Enclosed in each sporocyst, there are two sporozoites, comma-shaped cells that are the actual invading stages of coccidia parasites.

The oocyst wall is resistant to physical and chemical treatments like potassium dichromate 2% and sodium hypochlorite [13,14,15,16]. However, oocysts are sensitive to high temperatures and they are also permeable to lipophilic molecules; even though the wall is resistant to mechanical injuries, it is damaged due to abrasion and mechanical digestion inside the gizzard of a chicken and, as a result, sporocysts are released [17].

Once in the duodenum, the sporozoites emerge thanks to the action of bile salts and pancreatic enzymes like trypsin and chymotrypsin. These enzymes digest the Stieda and sub-Stieda bodies found on the anterior pole of sporocysts, which act as a barrier for the sporozoite [4,18]. Due to their continuous movement, sporozoites escape through the hole left by the Stieda body and they start invasion; throughout this process called excystation, the sporozoite consumes almost all its energy stored in the form of amylopectin granules in the cytoplasm [19].

The sporozoite is a sickle-shaped cell equipped with unique organelles that promote the entrance inside the host cell. The invasion process of *Eimeria* spp. is still poorly elucidated, but it is believed to be similar to other Apicomplexan parasites. It initially requires recognition and interaction of the sporozoite with the host cells; in vivo, *Eimeria* spp. infect with a high grade of specificity different tracts of the intestine (Table 1), while in vitro there is no cell specificity, since many cell lines can be infected by sporozoites (Table 2). This specificity might depend on unique conditions of the intestinal lumen, such as pH, enzymes, mucous, metabolites, etc., but the key mechanisms that regulate this behavior have not been elucidated yet [19]. Recognition of the target cells comes with the contact between sporozoite and cell surface molecules like glycoproteins, lectin receptors, and carbohydrate residues that act as receptors and ligands. Internalization is an active process that occurs through the participation of the organelles located on the anterior end of the sporozoite-like micronemes, the conoid, dense granules, and rhoptries. The conoid is the anchor point of the microtubules that grant support to the sporozoite and help the circular actin–myosin-dependent movement called *gliding*, used to enter cells. Starting from the conoid located on the apical tip, the sporozoite makes contact with the host cell all along its length, forming a moving junction through which the parasite propels itself into the host cell. This leads to the formation of a parasitophorous vacuole that encloses the sporozoite inside the cell cytoplasm. Micronemes are secretory organelles, rich in adhesive domains, and they are involved in many processes, including motility and invasion, by engaging a variety of host receptors and creating a strong bond between the host and the parasite’s membranes, called “moving junction” [19,20]. Rhoptry proteins are club-shaped organelles that are secreted during the early phase of invasion. They intervene by actively recruiting host proteins to the moving junction, but also after the invasion by providing the transfer of some products through the parasitophorous vacuole. Moreover, it is believed that the content of rhoptries and dense granules can reprogram cellular functions, such as ligand exposure and immune response [4,19].

Initial invasion *of E. acervulina, E. maxima, E. necatrix,* and *E. tenella* occurs in the villous epithelium, but they complete their endogenous development mainly in the crypts; the mechanism of translocation from villi to crypts is not fully understood, but it is believed to occur by intestinal immune cells [21].

Once in the host cell, the parasite undergoes its endogenous development. First the sporozoite enlarges and loses most of the apical complex and inner membrane; at this stage it is called trophozoite. Then, the next phase is schizogony, characterized by several rounds of asexual replication that lead to the formation of a multinucleated schizont, followed by the development of merozoites, the second invading stages of *Eimeria* spp. Merozoites share some characteristics with sporozoites, but they contain more micronemes and fewer rhoptry; their function is to increase the number of parasites in the gut in order to have a larger final yield of oocysts. After 3–4 rounds of schizogony, the last generation of merozoites initiates a single round of sexual replication, with the formation of gametes. *Eimeria* spp. develop two distinct stages: the macrogamete and the microgamete. Macrogametes are large cells characterized by polysaccharide granules used for energy storage and multiple wall-forming bodies, precursors of the oocyst wall [22]. Microgametes are small motile cells equipped with a pair of flagella that mediate their movement and fertilization of the macrogamete [22]. Fertilization of gametes leads to the formation of a wall-encased zygote, which is liberated in the gut and later excreted as unsporulated oocysts in the environment; from now on, the cycle starts again [4].

**Table 2:** Cell cultures used in *Eimeria* in vitro research.

Cell Type	*Eimeria* spp.	Stage of Development	Reference
Bovine kidney cells Avian fibroblasts.	*E. tenella*	Merozoites	[23]
cEF	*E. tenella*	Schizonts	[23]
HeLa, Human Amnion, PCK, cEF, mouse fiboblasts	*E. acervulina*	Trophozoites	[24]
PCK cells	*E. tenella*	Oocysts	[25,26,27,28]
PCK cells	*E. necatrix*	Merozoites	[29]
PCK cells	*E. brunetti*	Merozoites	[30]
PCK cells	*E. acervulina*	Schizonts	[31]
Caco-2, LMH, BHK, MDBK, HCT-8, VERO, MDCK, RK-13, IEC-6	*E. tenella*	Merozoites	[32]
cIECs	*E. tenella*	Merozoites	[33]
CLEC-213	Transgenic *E. tenella*	Gametes	[34]

## 3. In Vitro and In Ovo Methods to Study *Eimeria*

*Eimeria* life cycle comprises sequential steps of development that can be elucidated by the use of well-assessed in vitro models. The studies on the exogenous phase take into consideration the stages of oocyst, focusing on their viability and on the process of sporulation. The endogenous development is slightly harder to understand, and many different protocols have been assessed so far: most them require the use of cell lines that are able to support part of *Eimeria* development; others have been assessed also in ovo.

### 3.1. Sample Collection and Purification of Eimeria Stages

Propagation of *Eimeria* species in coccidia-free reared animals is an essential step in this field of research, and unfortunately, it is the only available method to obtain enough oocysts for in vitro research. The guidelines for propagation in chickens are well described by Shirley in *Guidelines on techniques in coccidiosis research* [14].

Before any in vitro assay, *Eimeria* oocysts need to be isolated and purified from stool or litter, and in some cases, directly from the cecal content of chickens [14]. The intestinal content can be collected either by scraping or by pepsin digestion of cecal cores (coalesced masses of oocysts) [14]. Next, the collected material, or the litter, can be homogenized in water and filtered with gauzes and sieves to remove the coarse fecal debris.

The subsequent step is flotation: oocysts can float in saturated salt or sucrose solutions, forming an opaque ring on the top of the container, where they can be easily collected [16]. The purified oocysts are usually preserved in potassium dichromate (2%), a strong oxidizing agent that allows oxygen exchange and prevention of bacterial colonization. Before in vitro invasion of cell cultures, oocysts undergo a cleaning step with sodium hypochlorite, which does not harm the viability and invading power of the internal sporozoites [13,14,15,16].

The collection and purification of sporozoites require some more steps: first the oocyst wall must be destroyed in order to obtain free sporocysts. In vitro, this step is successfully simulated by homogenizing the oocysts suspension with 0.5–1 mm glass beads in a tissue homogenizer [14]. Afterward, an enzymatic digestion step with pancreatic enzymes is applied to obtain sporozoites. A cocktail of 0.25% purified trypsin, chymotrypsin, and 0.5% bile salts (in particular, sodium taurocholate) has been identified as the best medium for excystation [35]. Sporocysts are incubated in the excystation medium for 60–90 min at 37–40 °C in order to then obtain a good yield of sporozoites [14].

At the end of the excystation process, the suspension will contain oocyst walls and unexcysted sporocysts that might interfere with the study, so a purification step is suggested. Schmatz and colleagues developed an efficient method to purify sporozoites with a diethylaminoethyl-cellulose (DE52) anion exchange chromatography [36]. They reported a purification efficiency of 94% of the total excysted for both *E. tenella* and *E. acervulina.* Also, the Percoll^®^ gradients technique has been investigated by Dulski and Turner [37]: according to their study, it is possible to purify an extensive amount of sporocysts and sporozoites in 50 and 55–60% Percoll^®^, respectively. However, both DE52 and Percoll^®^ rely on the use of reagents that might harm sporozoites. In addition, they are expensive and might be complicated to realize, so easy and cheap methods have been tested. Commercially available filter papers, cellulose filter support pad, or disposable Pluristrainer^®^ filters (5–10 µm) allowed sporozoites purification with good yields [38,39,40,41].

Some studies start from a merozoites inoculum, which can be recovered by infected tissues. Two main methods are indicated: Stotish and Wang described a purification protocol starting from ceca and the chorioallantoic membrane of chicken embryos [42]. In brief, five days after infection tissues are collected, cut in sections, and rinsed in phosphate-buffered Ringer’s solution with penicillin and streptomycin. Then they are digested for 30 min with a buffered solution containing bovine serum albumin 1 mg/mL and hyaluronidase 1 mg/mL. The merozoites in the supernatant are purified by several steps of centrifugation in 7.5% Ficoll and 10% Hypaque in Ringer’s solution and cleaned by filtration in a glass-bead column [42]. Another easy protocol that allows an increased final yield of *E. tenella* and *E. maxima* merozoites was also described [14]. The intestine/ceca of infected chickens is opened and cut in pieces and incubated in an excystation medium like the one described for sporozoites excystation at 41 °C for less than 30 min; an excessive time would result in merozoite destruction. The cleaned suspension is then filtered with either a 10 mm silk/cloth filter or a DE52 column [14]. Another protocol described the purification of schizonts and merozoites from ceca scrapings with Percoll^®^ 30% over a cushion of Percoll^®^ 50%. After a centrifuge at 17,000× *g* (15 min), the schizonts are distributed in the floating material, while merozoites gather in the red pellet at the bottom. The supernatant is diluted to obtain Percoll^®^ 25% and after another centrifuge, schizonts can be collected from the white pellet. Merozoites, instead, undergo a hypotonic shock step with distilled water in order to lyse the erythrocytes. This step lasts 30 s, followed by dilution with phosphate buffer to restore the tonicity. Then merozoites are washed and purified through a cotton wool plug [14].

So far, the purification of asexual stages has been described, but it can be helpful to isolate sexual stages, too. A procedure for *E. maxima* was described by Pugatsch et al. [43]: 138 h post infection the intestines of euthanized animals are washed and the mucosa is digested with hyaluronidase 0.5 mg/mL for 20 min. The mucosa is washed and filtered. Gametocytes are retained by 10 mm polymon filter and can be collected by centrifugation at 800× *g* for 5 min [43].

### 3.2. In Vitro Assessment of the Exogenous Phase

In vitro methods to understand the activity of a treatment of the exogenous phase are relatively simple, and the target of the study is usually the process of sporulation or the lytic power of some compounds.

Degeneration of oocysts can be defined by microscopic observation or by determining the quantity of lysed material by spectrophotometric analysis at 273 nm [44]. In some studies, oocysts were stained with propidium iodide, a fluorescent intercalating agent that is internalized only when the parasite’s wall is collapsed [45].

Sporulation index is defined as the number of sporulated oocysts of a treated group over the number of the control group; a lower sporulation index indicates lower pathogenicity of the parasite [46]. To measure it, a quantity of about 1–4 × 10^5^ fresh unsporulated oocysts is incubated in potassium dichromate 2.5% for 1–3 days with a treatment, then the percentage of sporulated and degenerated oocysts is determined by counting with a hemocytometer. The results of the treated oocysts are usually compared to a positive control like diclazuril, an anticoccidial drug known as delayer of sporulation [47]. With this protocol, new alternatives to classic coccidiostats have been studied recently: Gadelhaq et al. found that disinfectants like sodium hypochlorite, ethanol 70%, and formalin 10% have a strong power against sporulation, and some herbal extracts have also been proven to be effective (see Section 4.2.1). [46].

### 3.3. In Vitro Assessment of the Endogenous Phase

The key passages for *Eimeria* invasion development happen inside the host, so in vitro studies on cell cultures are fundamental.

Invasion and development assays test the ability of *Eimeria* sporozoites to invade a cell monolayer and to continue their development inside it. Invasion assays are powerful tools to screen potential anticoccidial compounds, to evaluate the pathogenicity of attenuated strains, and to understand the molecular mechanisms at the basis of the process. Moreover, some protocols allow the propagation of low doses of oocysts in vitro [26,27]. Many different cell lines have been used so far to study *Eimeria* spp. in vitro (Table 2).

The early attempts to cultivate *Eimeria* spp. started in 1965 when Patton first identified the cell types able to be invaded by *E. tenella* sporozoites and to allow development at least up to schizonts. Bovine kidney epithelial cells, cultured at 41 °C, resulted in the most suitable cell culture [23]. A similar study was done on *E. acervulina* by Strout et al.: they found that the parasite could develop up to trophozoites in various cell lines (HeLa, human amnion, primary kidney epithelial cells, chicken embryonic fibroblasts, mouse fibroblasts), confirming the previously described lack of specificity in vitro [24]. A further study on *E. tenella* was done by Doran (1970); he managed to infest epithelial kidney chicken cells (PCKs) in vitro and to obtain oocysts by re-infection of a new culture with second generation merozoites [25]. PCK cells appear to be the best model for *Eimeria* research and they have been extensively used in research so far [14]. PCK cells allow a good degree of development for *E. tenella,* and therefore they could be used to compare a precocious strain with a wild-type one: it was highlighted that the precocious strain entered the sexual phase, bypassing the second generation of schizogony 96 h post-infection with sporozoites [48]. In optimal culture conditions, new oocysts can be produced in vitro: Hofmann and Raether observed an increased formation of oocysts in PCKs cultured on commercial calf collagen-coated plates, but the oocyst yield was still very low and the morphology was compromised [26]. Zhang et al. propagated *E. tenella* with relatively good yields: they underlined the importance of regular medium change (RPMI 1640 supplemented with 10% fetal bovine serum) and appropriate culture supports (coverslips on a 24-well plate), and they also adapted a field strain to grow in PCK cultures by multiple passages from host to cells; hence, they got three times more oocysts than the previous studies [27,28]. From these results, it is clear that oocysts can be obtained in cell cultures, thus highlighting the potentiality of this tool for in vitro development studies; however, propagation inside the live host is not replaceable: organ specificity of the coccidia, absence of gastrointestinal factors, and a poor adaptation of the parasite to in vitro culture might be the reasons for the low quality and insufficient number of oocysts yield in vitro [26]. PCK cultures have also been used to study other species of *Eimeria*; second generation merozoites have been obtained in culture starting from *E. brunetti* and *E. necatrix* sporozoites [29,30]. *E. acervulina* has been cultivated up to first generation schizonts, but zygote formation was observed when the inoculation sample contained merozoites isolated from infected animals [31]. PCK cultures are not immortalized and they need to be isolated before every assay, thus involving the sacrifice of animals. In addition, the culture medium needs to be adapted to the culture phase, otherwise the cells peel and form clusters. Furthermore, the sporozoite suspension needs to be extremely pure because oocyst walls, unexcysted sporocysts, and residual debris have a toxic effect on the cells [26]. PCK cultures are species-specific and they provide an excellent substrate to investigate *Eimeria* development in vitro, but due to the abovementioned factors, the latest studies have been done on immortalized cells, like Madin Darby Bovine Kidney (MDBK) cells. A comparative study of *E. tenella* development in different cell lines was done by Tierney and Mulcahy (2003); they proved that MDBK cells were the best epithelial lines for supporting the parasite’s development up to merozoites. In their study, temperature was fundamental for the parasite’s growth: cells growing at 41 °C instead of 37 °C supported the highest degrees of development [32]. In MDBK cells, *Eimeria* sporozoites develop into fully formed first generation merozoites; however, these do not reinvade new MDBK cells, for reasons that remain unclear [49].

A recent study conducted by Bussière et al. established that a chicken lung epithelial cell line (CLEC-213) is able to support *Eimeria* growth up to second generation merozoites. Infecting CLEC-213 with merozoites, gametes were found in culture [34]. Many cell lines have been tested so far, creating divergences in methods and results. A reproducible in vitro method to cultivate *Eimeria* in intestinal epithelial cells, a natural target of the parasite, is still lacking due to the difficulty of obtaining these cells and culturing them in vitro. An early attempt was made by Dimier-Poisson et al. (2004). They managed to isolate chicken intestinal epithelial cells (cIECs) from d18 chicken embryos. After 60 h from seeding, the cells were infected with *E. tenella* sporozoites. With this method they kept them in culture for over 40 h, when merozoites were visible, but no further development occurred [33]. However, this study did not report any detailed information about the cell line characterization, their survivability, and their polarization, intended as the ability to form junctions and arrange themselves, creating an apical and basolateral side, which is a fundamental feature of intestinal cell models [50].

### 3.4. In Ovo Culture

**Figure 2:**
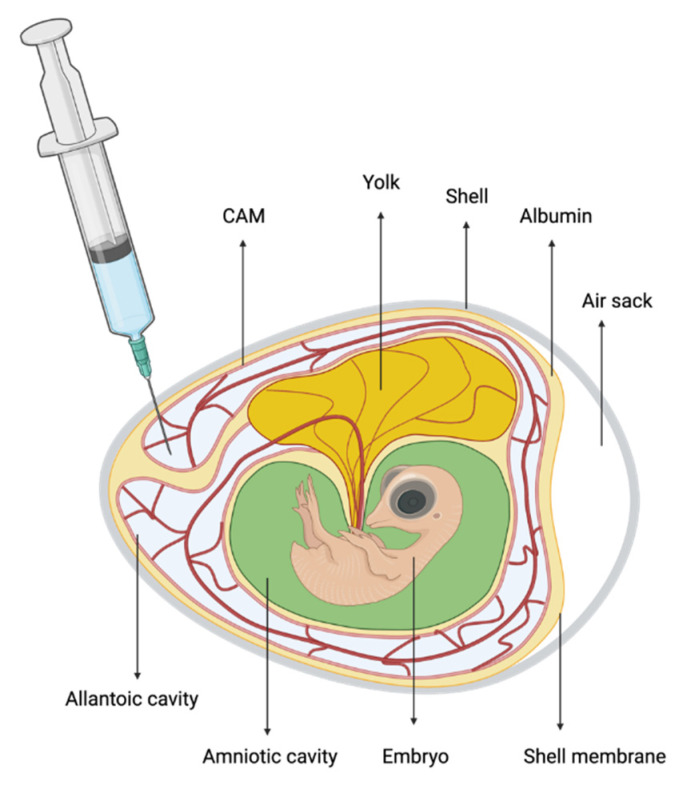
Schematic representation of an embryonated egg model. The syringe shows the site of inoculation preferred for *Eimeria* research.

Infection of the host normally occurs by ingestion of sporulated oocysts and development of the species is usually confined to the intestine; however, embryonated eggs have been widely used as a substrate for *Eimeria* replication, especially for attenuated vaccine production [51]. In 1965, Long demonstrated that the development of *E. tenella* can occur at a site other than the caeca, such as the chorioallantoic membranes (CAMs) of chicken embryos. In that study, different sites of injection were tried, such as the intravenous route, amniotic route, and allantois, with different dosages of sporozoites. The best results occurred for the allantois injection (Figure 2) with 6.4 × 10^4^ sporozoites: the CAMs of all embryos proved positive for parasites when examined between days 4 and 11 of infection with visible schizonts, gametocytes and oocysts, respectively, from days 4 and 7; and the mortality found was low. In preliminary experiments, deaths occurred in high numbers up to 24 h post injection for heavy bacterial contamination. To control this, prior to excystation, the oocysts were accurately washed and sterilized with penicillin and streptomycin (50 IU/mL), 1:500 Chloros solution, and 2% formalin solution [52]. A second study by Long (1966) assessed the infection of chicken embryos with *E. tenella, E. necatrix, E. brunetti, E. mivati, E. acervulina,* and *E. maxima* [53]. Among these species, *E. brunetti, E. mivati,* and *E. tenella* could complete the entire cycle up to oocysts; *E. necatrix* developed only to late schizogony, and *E. acervulina* and *E. maxima* did not develop. The life cycles were delayed compared to the in vivo natural cycle, but the oocysts recovered from the CAMs sporulated normally and induced infections in chickens, meaning that the development generated normal oocysts and it could represent a reliable tool for in vitro research [53].

This tool was also used to produce attenuated strains of *Eimeria* by passage in chicken embryos. In 1972, Long successfully obtained a less pathogenic strain of *E. tenella* through 42 passages in embryos [54]. The mechanism of this adaptation was explained as a selection of schizonts or merozoites that are capable of completing their life cycle in the embryo chorioallantois. In further studies, the loss of pathogenicity was noted to occur concurrently with the appearance of smaller lesions and the absence of large second generation schizonts both in ovo and in vivo [55].

The discovery that sporozoites of *Eimeria* would colonize chicken embryos led to the development of in vitro assays to test anticoccidial drugs. In 1970 the effect of several anticoccidials was examined against three strains of *E. tenella*. The drug effect was assessed by mortality on day 3 after sporozoite injection and by foci count, associated with colonies of second generation schizonts on the CAM. Also, two routes of administration were assessed: inoculation into the allantois at day 10 (one day before sporozoite injection, which occurred at day 11) and inoculation into the yolk sac on day 4 for lower absorption. The method was successful in finding the resistance of some strains to some drugs, to screen the efficacy dose, and to highlight that some drugs are more effective when inoculated by the yolk sac route. Moreover, the counting of foci appeared to be a sensitive method to assess anticoccidial efficacy and preferable to mortality as a criterion [56]. In 1990, Ball and colleagues infected chicken embryos with a reduced dose of sporozoites (1000) to monitor oocyst production with and without treatment with lasalocid, via administration in the allantoic cavity [57]. Even though the anticoccidial did not prevent death from coccidiosis, the oocyst production was significantly lower for the highest dose tested.

In 1991, Xie and colleagues attempted to screen the anticoccidial activity of some drugs in the CAMs of 11 d old embryos [58]. To prevent mortality due to coccidiosis an attempt at infection for a shorter period (60 h) with second generation merozoites was also made, and the production of oocysts was taken as a parameter to assess effectiveness; since the results disagreed with literature, the authors concluded that the embryo model is limited for the evaluation of unknown compounds [58]. However, embryos provide an uncontaminated environment for *Eimeria* spp. to complete their life cycle and to collect merozoites, gametes, and oocysts in good yields and also provide important biochemical and immunological information [59]. Embryonated chicken eggs were successfully used as a model for a co-infection with *Clostridium perfringens* and *E. tenella*, major agents involved in necrotic enteritis. *E. tenella* was inoculated via the allantoic sac route at day 10, while *C. perfringens* was inoculated at day 15. The CAM was removed from the egg shell for macroscopic lesion scoring, histological examination, and for DNA quantification by quantitative polymerase chain reaction (qPCR). The gross lesions scoring was determined in a range from 0 to 3 (Table 3). Lesions were demonstrated in histological sections of the infected groups: *Eimeria* schizonts were present in the form of white foci in the CAM; also, necrosis and leukocyte infiltration were apparent at the endomesoderm contacting the allantoic fluid (AF). For the molecular quantification of *Eimeria* DNA, the oocysts were isolated from the AF and the CAM after homogenization of the tissue with glass beads. Then, DNA was extracted and a fragment of the ITS1 region of *E. tenella* was amplified by qPCR. By their observations, Alnassan and colleagues concluded that *Eimeria* infections create an advantageous environment for *C. perfringens* multiplication. This study may open the way to a broad variety of in vitro studies, i.e., host–pathogen interaction, inter-pathogen interaction, and immunopathological studies and research on the effect of pharmaceutical compounds on mixed infections [60].

## 4. In Vitro Assessment of Conventional and Alternative Anticoccidials

In the previous section, the primary tools to study *Eimeria* behavior in vitro were presented. These means have been extensively used to understand the mechanisms of action of the available treatments against coccidiosis. In the following paragraphs, the main findings obtained by the application of the presented methods will be reviewed, focusing on ionophores and synthetic compounds, as well as on botanicals with anticoccidial action.

### 4.1. Ionophores and Synthetic Compounds

Despite the adoption of strict hygiene and sanitation measures, eradication of coccidiosis in flocks has not been possible, so by now, pharmaceutical treatments and immunization involving the use of attenuated and non-attenuated vaccines are the main means to control this pathology [5]. As treatment after the onset of clinical signs is often too late to prevent the consequences of infection, prophylaxis has been preferred ever since the introduction of sulfaquinoxaline. Nowadays, anticoccidial drugs belong to two major categories: ionophores and synthetic compounds and many have been tested in vitro (Table 4) [5].

Ionophores (monensin, lasalocid, salinomycin, narasin, and maduramicin) are produced by fermentation of bacterial species and are widely used because resistance develops slowly and a small part of parasites develop, thus permitting immunity response [5]. They disturb the normal transport of ions across the surface of sporozoites and trophozoites; also, most foodborne bacteria are not disturbed by these molecules. Ionophores are therefore not included in the WHO list of medically important antimicrobials [5]. They are categorized in two classes: monovalent ionophores, which can form lipid-soluble complexes with sodium and potassium ions, and divalent ionophores, which can bind magnesium and potassium. Also, ionophores can significantly increase the intracellular Na+ concentration in sporozoites, thus boosting the activity of Na+-K+-ATPase and use of ATP [61]. Scintillation studies by Smith and Strout in 1979 showed that free sporozoites can incorporate and retain ionophores, suggesting that the accumulation of the drug is independent from the host cell and that ionophores are more effective when administered prior to infection (this explains why the therapeutic power of these compounds is very low compared to the prophylactic one) [62]. Ultrastructural studies by electron microscopy showed that sporozoites incubated with ionophores are subject to morphological abnormalities, such as swelling on the anterior pole, suggesting that the anticoccidial effect happens by osmotic disruption. This behavior was observed by Melhorn et al. in free sporozoites and merozoites after 30 min of incubation with different concentrations of ionophores (monensin, salinomycin, and lasalocid) ranging between 1 and 1000 µg/mL [63]. In addition, transmission electron microscopy observation of invaded PCK cells showed the same irregularities in intracellular sporozoites treated with 0.1 µg/mL of narasin and monensin simultaneously with infection [64].

McDougald and Galloway suggested that sporozoite invasion of the host cell is also a necessary prerequisite for the anticoccidial activity of ionophores [65]. Smith et al. observed that ionophores could limit the invasion of sporozoites in PCK cells and intracellular development up to schizonts to a major extent; they suggested that unspecified changes occur in the parasite within the host cell and may enhance the anticoccidial proprieties of ionophores [66].

Also, ionophores appear to be very effective on free merozoites, which are killed more rapidly compared to sporozoites, advising that ionophores should be fed daily to maximize the effect on all the endogenous stages [63].

The efficacy of ionophores on *Eimeria* spp. development was further investigated in ovo: Long and Jeffers (1982), and later Ball et al. (1990), observed improvements in embryo mortality, specific lesions, hemorrhages in the CAM and oocyst count and viability in embryos challenged with ionophore-treated sporozoites [57,67]. In 1991, Xie and colleagues attempted to screen the anticoccidial activity of some drugs in the CAM of 11 d old embryos [58]. Both the minimal inhibitory concentration (MIC) and minimal toxic concentration (MTC) of the compounds were assessed: the MIC was declared as the lowest level tested that prevented mortality, while the MTC was the lowest concentration that caused toxic death (deaths without signs of coccidiosis); however, in their study these two parameters usually matched, and some compounds gave misleading results, so this led to the conclusion that the embryo model as well as cell cultures are often limited for the evaluation of unknown compounds, as the dosage and endpoint of activity can differ a lot from the in vivo situation, and in some cases a compound needs to be metabolized by the animal to the active form [58].

However, the positive aspects of in vitro should not be disregarded, especially in the scenario of anticoccidial resistance. In fact, in vitro assays have also been used to detect traits of resistance/sensitivity to ionophores: in a study by Augustine et al. an ionophore-sensitive strain of *E. tenella* was compared to an ionophore-resistant one isolated from the field. They incubated sporozoites of the two strains with different concentrations of ionophores in PCK cells for 72–96 h in order to count the intracellular developmental stages; in addition, they measured the differential uptake of radio-labeled monensin. They documented that the resistant strain of *E. tenella* was characterized by a reduced uptake of the drug, and the amount required to inhibit *E. tenella* invasion and development in PCK cells is 40 times lower in ionophore-sensitive strains [68]. With the advent of molecular methods, qPCR has been increasingly used to study coccidia: Jenkins infected MDBK cells with either a resistant or a sensitive strain of *E. tenella* sporozoites in the presence of different concentrations of ionophores; after 24 h, cells were fixed for intracellular counting or harvested for qPCR. By these methods, it was possible to build a curve of sensitivity to the tested ionophores for both strains [39]. A further step was done by Thabet et al.: MDBK cells were infected with *E. tenella* sporozoites, and by qPCR they quantified the percentage of sporozoite invasion and the percentage of reproduction inhibition 96 h post-infection, documenting an in vitro MIC for a pool of ionophore compounds [9]. This study opened the way to carry on anticoccidial efficacy testing with the significant advance of rapid, objective, standardized, and cost-effective methods. Recently, qPCR was used to monitor *E. tenella* growth in MDBK cells after treatment with a pool of anticoccidials, among which salinomycin was proven to inhibit significantly the parasite intracellular development [49].

Synthetic compounds (nicarbazin, amprolium, zoalene, decoquinate, clopidol, robenidine, and diclazuril) are produced by chemical synthesis and there are three main known modes of action.

Inhibition of mitochondrial respiration (decoquinate and clopidol): these drugs exploit the fact that *Eimeria* spp. respiration differs from vertebrates [5]. They inhibit sporulation and early development in cell cultures by blocking the mitochondrial respiration, as seen in in vitro assays [9,69,70,71]. The action of these compounds is often reversible: in fact, they act by inhibiting coccidia rather than killing them, and the target is thought to lie between coenzyme Q and cytochrome B of the mitochondrial respiration. Due to the lack of cross resistance, the mechanism of action of compounds of this class is thought to be slightly different among them, so they are often used in combination. However, this issue still needs to be elucidated in detail [5,69].

Inhibition of folic acid pathway (sulfonamides and ethopabate): they interfere with the synthesis of folic acid by competitive antagonism, impeding the synthesis of nucleic acids and cellular division in *Eimeria* spp., characterized by high demands of this compound; however, these are rarely used in broiler production because of the high potential for residues and lack of activity against certain species [5]. In vitro studies for these compounds are not very common: McDougald and Galloway observed that sulfamethazine could delay *E. tenella* development in PCK cells as long as the compound was in culture, but then development restarted, suggesting a reversible action of this compound [70].

Competitive inhibition of thiamine uptake (amprolium): amprolium hydrochloride is an analogue of thiamine and so it inhibits its uptake by second generation schizonts [72]. The formation of thiamine phosphate, which is essential for many biochemical reactions, such as the activity of decarboxylases, is then hindered [5]. Amprolium was minimally tested in vitro: McDougald and Galloway observed an inhibitory effect on schizogony during a development assay in PCK cells, which was present even after withdrawal of the compound [70]. In vitro no efficacy was observed on MDBK invasion assays and, in ovo, Xie et al. observed that amprolium had very little efficacy [49,58].

Unspecified mode of action: for some compounds, it was not possible to identify a mode of action. Nicarbazin is one of the first synthetic compounds with a broad spectrum of activity; however, it is not indicated in summer, because it can increase the risk of heat stress, and in laying hens it negatively affects egg production [5,73]. The mode of action seems to occur by inhibition of succinate and ATP transhydrogenases and accumulation of intracellular calcium in second generation schizonts [74]. Diclazuril is a nucleoside analogue, and it is thought to affect later stages of development [5]. By reverse transcription qPCR, it was shown to downregulate microneme proteins and serine/threonine protein phosphatase expression in *E. tenella* merozoites isolated from chicken ceca [75]. Robenidine is a derivate of guanidine that prevents formation of first generation schizonts as observed in vitro, also after withdrawal in PCK cell culture [70]. Marugan-Hernandez et al. also documented a significant reduction in sporozoite invasion in MDBK cells by detection of the intracellular *E. tenella* genome by qPCR [49]. Even though the exact mode of action is unknown, it seems to be related to inhibition of energy metabolism [5].

Many cases of resistance have arisen to all of these compounds [76]. The life cycle of *Eimeria* spp. includes haploid asexual stages, and if a resistant-mutant appears it is rapidly selected in the presence of the drug, and as coccidia heavily multiply inside the intestine, the resistant traits become the dominant phenotype [76]. Resistance to ionophores develops slowly in the field, as destruction of the parasite occurs very early: as stated by Augustine et al. a difference in uptake might be related to comparison of resistance toward ionophores, but the mechanisms are not well understood, though they may be due to alteration of their cell membrane [68,77]. Sulfonamide-resistance cases have been associated with mutation in two genes: the dihydrofolate reductase (*dhfr*) and the dihydropteroate synthase (DHPS). Concerning amprolium and mitochondrial respiration inhibitors, as the mechanism of action relies on inhibition of enzymatic activity, resistance is thought to occur by alternative biochemical pathways [76,77].

**Table 4:** Main anticoccidial drugs and relative mode of action and target.

Anticoccidial Drug	Mode of Action	Target Stage of *Eimeria* spp.	*Eimeria* spp.	Lowest Effective Concentration In Vitro	In Vitro Test	Reference
Amprolium	Inhibition of thiamine uptake	Second generation schizonts	*E. tenella*	4 µg/mL	Development assay	[70]
Clopidol	Inhibition of mitochondrial respiration	Sporozoites and sporulation	*E. tenella*	4 µg/mL	Development assay	[70]
Decoquinate	Inhibition of mitochondrial respiration	Sporozoites and sporulation	*E. tenella*	0.01 µg/mL	Development assay	[65]
Diclazuril	Nucleoside analogue	Late stages of development	*E. tenella*	0.125 µg/mL	Sporulation assay	[47]
Lasalocid	Ionophore	Sporozoite and trophozoite	Various spp.	0.5 µg/mL	Invasion and development assay	[9]
Monensin	Ionophore	Sporozoite and trophozoite	Various spp.	0.001 µg/mL	Development assay	[65]
Narasin	Ionophore	Sporozoite and trophozoite	Various spp.	0.01 µg/mL	Electron microscopy	[64]
				1 µg/mL	Invasion and development assay	[66]
Nicarbazin	Inhibition of succinate dehydrogenase and accumulation of intracellular calcium	Schizonts	Various spp.	4 µg/mL	Development assay	[70]
Robenidine	Guanidine derivate	Schizonts	Various spp.	4 µg/mL	Development assay	[70]
Salinomycin	Ionophore	Sporozoite and trophozoite	Various spp.	0.1 µg/mL	Invasion and development assay	[66]
Toltrazuril	Inhibition of mitochondrial respiration	Sporozoites, schizonts and gametes	Various spp.	5 µg/mL	Invasion and development assay	[9]

### 4.2. Botanicals with Anticoccidial Efficacy In Vitro

#### 4.2.1. Botanicals that Target the Exogenous Phase of *Eimeria* spp.

Many botanicals that target the exogenous phase have been tested in vitro (Table 5). Artemisia, thyme, clove, and tea tree oils were analyzed in vitro by Remmal et al.: they saw that an incubation for 20 h in media containing the cited EO led to oocyst disruption and a 5-fold decrease of oocyst number [78]. A pure product extracted from *Artemisia annua,* artemisinin, was also tested by del Cacho and colleagues (2010). They found a dose-dependent increase of dead oocysts shed in feces, an alteration in the sporulation rate, and a significant reduction of calcium ATPase in macrogamete endoplasmatic reticulum, which most likely leads to abnormal oocyst formation [45]. Green tea from *Camellia sinensis*, rich in selenium and polyphenols, was tested by Molan and Faraj on three species of *Eimeria* (*E. tenella, E. acervulina,* and *E. maxima*): they found a consistent impairment of sporulation in parasites incubated with 10 and 25% of tea extract [79]. Another study by Jitiriyanon et al. evaluated the in vitro anticoccidial proprieties of various EOs in terms of sporulation. They found that *Boesenbergia pandurata* and *Ocimum basilicum* EOs were very effective and that methyl cinnamate, camphor, and methyl chavicol were their active components [80]. A significant inhibition of sporulation was also documented for *Prangos ferulacea, Artemisia absinthium, Biarum bovei, Nectaroscorum tripedale,* and *Dorema aucheri* by Habibi et al. and for *Pinus radiata* extract by Molan et al. on various species of *Eimeria* [81,82]. The mechanism of EO on sporozoites might be related to rapid diffusion through the parasite membrane; loss of permeability and leakage of ions follows, leading to cell death. Pomace is a promising source of valuable substances, as it contains numerous bioactive compounds, so the anticoccidial power of olive pulp and standard quercetin and oleuropein was shown by Debbou-Iouknane by oocyst lysis [44]. Curcumin is a natural polyphenolic compound found in *Curcuma longa,* commonly used as a medicinal herb, and garlic is considered as one of the most essential and effective herbs for medicinal purposes: El-Khtam et al. documented an effect on sporulation for turmeric and garlic powder, which was inhibited by 66 and 80%, respectively, at the highest concentration (10 g/L) [83].

#### 4.2.2. Botanicals that Target the Invasion of *Eimeria* spp.

Many botanicals that target the endogenous phase have been tested in vitro (Table 6).

Khalafalla et al. reported considerable alterations in sporozoite morphology that affected the parasite viability and infectivity during an invasion assay in MDBK up to 72% for curcumin [84]. Curcumin was also tested by Burt et al. (2013) with other phytochemicals: carvacrol, a major constituent of oregano, and *Echinacea purpurea* extract, known for its immunomodulatory activity [85]. The influence of these compounds was observed by invasion assay on MDBK cells; the invading capability of *E. tenella* was inhibited by curcumin, carvacrol, and *E. purpurea* extract, more effectively when these compounds were used in combination, thus highlighting a synergistic effect [85]. One of the main issues of working with natural extracts lies on the wide variability of their composition, which is deeply influenced by a lot of factors like plant characteristics and methods of extraction. In order to exclude this limitation and to obtain reproducible results, choosing nature-identical compounds is often preferable [86]. In a recent study, Felici et al. proved the inhibiting activity of chemically synthetized thymol and carvacrol, major constituents of thyme, on invasion on MDBK cells. The efficacy of thymol and carvacrol-based treatment was improved in synergy with saponins, pointing up, once again, the synergistic potential of botanical molecules [87]. A similar study was performed by Sidiropoulou et al. with EOs; oregano and garlic EO pretreatment on sporozoites resulted in a strong anticoccidial activity in vitro, as well as a positive result on intestinal microbiota and growth performance in vivo [88]. Garlic is another important source of anticoccidial compounds and some garlic extracts like propyl thiosulfinate, propyl thiosulfinate oxide, and allicin have been shown to have anticoccidial efficacy. Alnassan et al. reported a steep decrease in intracellular *E. tenella* DNA after invasion in MDBK cells treated with various concentrations of allicin, with reductions ranging from 54% (for 1.8 ng/mL) up to 99% (for 180 mg/mL) [89]. An alternative to anticoccidial treatments can also be provided by *Thonningia sanguinea*, an African medical plant that was shown to have a significant inhibitory effect on invasion for *E. tenella* and *E. necatrix* by Séverin et al. in MDBK cells [90].

**Table 6:** Botanicals whose action on the endogenous phase has been assessed by in vitro invasion assays (essential oil, EO; Madin Darby Bovine Kidney, MDBK).

Compound	Concentration	*Eimeria* spp.	Method	Reference
Allicin	1.8 × 10^−3^–1.8 × 10^3^ µg/mL	*E. tenella*	Infection on MDBK and qPCR detection	[89]
Betaine	0.5 µg/mL	*E. tenella*	Infection on MDBK and count in HE stain	[85]
Carvacrol	20 µg/mL	*E. tenella*	Infection on MDBK and count in HE stain	[85]
Curcumin	73.6 µg/mL	*E. tenella*	Infection on MDBK and flow cytometry quantification	[84]
Curcumin	0.2 µg/mL	*E. tenella*	Infection on MDBK and count in HE stain	[85]
Echinacea purpurea extract	2 µg/mL	*E. tenella*	Infection on MDBK and count in HE stain	[85]
Garlic EO	50 µg/mL	*E. tenella*	Infection on MDBK and qPCR detection	[88]
Oregano EO	100 µg/mL	*E. tenella*	Infection on MDBK and qPCR detection	[88]
Saponins	10 µg/mL	*E. tenella*, *E. acervulina*, *E. brunetti*	Infection on MDBK and extracellular counts and qPCR	[87]
Thymol and carvacrol	14 µg/mL	*E. tenella*, *E. acervulina*, *E. brunetti*	Infection on MDBK and extracellular counts and qPCR	[87]
Thonningia sanguinea extract	625–40,000 µg/mL	*E. tenella*, *E. necatrix*	Infection on MDBK and counts Giemsa stain	[90]

## 5. Conclusions

Coccidiosis is a widespread disease affecting avian species. The causative agents are *Eimeria* spp., protozoan parasites characterized by an intricate life cycle difficult to study in vitro. Many protocols have been used to monitor the various stages of *Eimeria*; however, results are sometimes contrasting and a gold standard is still missing.

This review aims to recapitulate the benefits and limitations of in vitro models to study coccidia and to screen anticoccidial molecules by describing their modes of action. Many studies focus on the exogenous phase of the parasite, considering indexes such as sporulation rate and viability of oocysts as good indicators to predict the efficacy of a molecule.

However, most of the *Eimeria* life cycle occurs inside the host, so a full understanding cannot disregard cell infection in vitro. Invasion and development assays have been attempted for decades on several cell lines, and, contrary to what happens in vivo, many resulted in being permissive to sporozoites, PCKs and MDBK cells being the best candidates so far, although chicken intestinal epithelial cells (cIECs) would represent a closer model to the natural target of *Eimeria*. In ovo protocols have been successfully used to study *Eimeria* spp. and the efficacy of anticoccidials: sporozoites and merozoites can complete their life cycle in the CAMs of chicken embryos, producing characteristic lesions that can be taken into account as good parameters of infection, in combination with embryo mortality, oocyst production, and molecular quantification methods.

Many discoveries in invasion, development, and biochemical processes would not be possible without well-assessed in vitro methods. These protocols allow screening the anticoccidial efficacy of both conventional and new molecules successfully, reducing costs, time, and animal sacrifice.

The demand of new compounds is increasing remarkably, and it is important to identify new candidates against coccidiosis and to understand their target and mechanism of action. Treatments of the exogenous phase find their implementation in the disinfection and cleaning procedures of the litter in farms. EOs can improve the hygiene level in the production facilities as an alternative to chemical agents; however, the concentration of these blends is often elevated and this could increase the costs and make the handling more complicated [91]. Lower concentrations are used in the framework of the endogenous phase, meaning that anticoccidial treatments are more effective when administered to the host and find their target in the invading and developmental stages of *Eimeria* spp. More often, nature-identical compounds are tested in vitro against invasion and development rather the relative EOs: this reliably prevents the inconvenience of variable compositions and lack of standardization. A well-defined formulation and mode of action are urgently needed in the development of feed additives [86].

In vitro invasion and development assays, along with sporulation rate and oocyst lysis measurements, can all provide useful insights on the mechanisms of action of molecules. Nevertheless, in vivo research still remains the endpoint to assess the impact of coccidia in an organism as in vitro models do not accurately translate into a whole organism. Regardless, implementing fast and relatively cheap in vitro technologies allows a better understanding of coccidia pathogenesis and helps to screen anticoccidial molecules.

## Figures and Tables

**Table 1:** Main *Eimeria* spp. infecting chickens with their relative site of infection and level of lesion severity (adapted from [4]).

Species	Site	Pathogenicity
*E. acervulina*	Upper small intestine	Medium
*E. brunetti*	Distal small intestine and colon	High
*E. maxima*	Mid small intestine	Medium
*E. mitis*	Upper small intestine	Low
*E. necatrix*	Mid small intestine	High
*E. tenella*	Ceca	High

**Table 3:** Main indicators to assess the severity of *Eimeria* infections in ovo.

Method	Pathogen	Method Description	Reference
Embryo mortality	*E. tenella*	Percentage of dead embryos 3 days post inoculation was considered as caused by *Eimeria* spp.	[58]
Oocyst production	*E. tenella*	Oocyst count in the allantoic cavities at day 7 post-infection	[57]
Schizont dimension	*E. tenella* (egg-adapted)	Absence of large second generation schizonts in the CAM is linked to loss in pathogenicity	[55]
CAM foci count	*E. tenella*	Count of foci corresponding to second generation schizonts on the CAM	[56]
Lesion scoring	*E. tenella* and *C. perfringens* dual infection	0 = no lesion 1 = hemorrhage and congestion 2 = fewer than 10 white foci per cm^2^ 3 = 10 or more white foci per cm^3^ plus hemorrhage and congestion	[60]
Histological observation	*E. tenella* and *C. perfringens* dual infection	Yellow inflammatory foci containing necrotic material and leukocytic infiltration on the CAM	[60]
qPCR	*E. tenella*	Absolute DNA quantification by a plasmid containing ITS1 region insert. The *Eimeria* sample was collected from the CAMs and AF of chicken embryos	[60]

**Table 5:** Botanicals whose action on the exogenous phase has been assessed by in vitro methods (essential oil, EO).

Compound	Concentration	*Eimeria* spp.	Method	Reference
Allium sativum	10 g/L	*E. tenella*, *E. maxima*, *E. acervulina*, *E. necatrix*, *E. mitis*	Sporulation rate	[83]
Artemisia EO	4 g/L	*E. tenella*, *E. maxima*, *E. acervulina*, *E. necatrix*, *E. mitis*	273 nm absorbance	[78]
Artemisia absinthium EO	50 g/L	*E. tenella*	Sporulation rate	[81]
Artemisinin	0.01–0.017 g/L	*E. tenella*	Sporulation rate	[45]
Boesenbergia pandurate EO	0.125 g/L	*E. tenella*	Sporulation rate	[80]
EOBiarum bovei EO	50 g/L	*E. tenella*	Sporulation rate	[81]
Clove EO	4 g/L	*E. tenella*, *E. maxima*, *E. acervulina*, *E. necatrix*, *E. mitis*	273 nm absorbance	[78]
Curcumin	10 g/L	*E. tenella*, *E. maxima*, *E. acervulina*, *E. necatrix*, *E. mitis*	Sporulation rate	[83]
Dorema aucheri EO	50 g/L	*E. tenella*	Sporulation rate	[81]
Nectaroscorum tripedale EO	50 g/L	*E. tenella*	Sporulation rate	[81]
Ocimum basilicum EO	0.125 g/L	*E. tenella*	Sporulation rate	[80]
Olive pulp	0.023–0.371 g/L	*E. acervulina*, *E. tenella*, *E. mitis*, *E. brunetti*, *E. maxima*	273 nm absorbance	[44]
Prangos ferulacea, EO	50 g/L	*E. tenella*	Sporulation rate	[81]
Pinus radiata extract	0.250–1 g/L	*E. tenella*, *E. maxima*, *E. acervulina*	Sporulation rate	[82]
Thyme EO	4 g/L	*E. tenella*, *E. maxima*, *E. acervulina*, *E. necatrix*, *E. mitis*	273 nm absorbance	[78]
Tea tree EO	4 g/L	*E. tenella*, *E. maxima*, *E. acervulina*, *E. necatrix*, *E. mitis*	273 nm absorbance	[78]
Green tea extract (Camellia sinensin)	100 g/L	*E. tenella*, *E. maxima*, *E. acervulina*	Sporulation rate	[79]

## Data Availability

All references found eligible in our literature review are included in the article.

## Abbreviations

NICs	nature-identical compounds
EO	essential oil
PCKs	primary kidney chicken cells
MDBK	Madin Darby Bovine Kidney
CLEC-213	chicken lungs epithelial cell line
cIECs	chicken intestinal epithelial cells
DE52	diethylaminoethyl-cellulose
CAM	chorioallantoic membrane
AF	allantoic fluid
qPCR	quantitative polymerase chain reaction
MIC	minimal inhibitory concentration
MTC	minimal toxic concentration
dhfr	dihydrofolate reductase
DHPS	dihydropteroate synthase
